# The bZIP transcription factor HY5 interacts with the promoter of the monoterpene synthase gene *QH6* in modulating its rhythmic expression

**DOI:** 10.3389/fpls.2015.00304

**Published:** 2015-04-30

**Authors:** Fei Zhou, Tian-Hu Sun, Lei Zhao, Xi-Wu Pan, Shan Lu

**Affiliations:** State Key Laboratory of Pharmaceutical Biotechnology, School of Life Sciences, Nanjing UniversityNanjing, China

**Keywords:** *Artemisia annua*, circadian rhythm, HY5, monoterpene synthase, *QH6*, G-box

## Abstract

The *Artemisia annua* L. β-pinene synthase QH6 was previously determined to be circadian-regulated at the transcriptional level, showing a rhythmic fluctuation of steady-state transcript abundances. Here we isolated both the genomic sequence and upstream promoter region of *QH6*. Different regulatory elements, such as G-box (TGACACGTGGCA, −421 bp from the translation initiation site) which might have effects on rhythmic gene expression, were found. Using the yeast one-hybrid and electrophoretic mobility shift assay (EMSA), we confirmed that the bZIP transcription factor HY5 binds to this motif of *QH6*. Studies with promoter truncations before and after this motif suggested that this G-box was important for the diurnal fluctuation of the transgenic β-glucuronidase gene (*GUS*) transcript abundance in *Arabidopsis thaliana*. *GUS* gene driven by the promoter region immediately after G-box showed an arrhythmic expression in both light/dark (LD) and constant dark (DD) conditions, whereas the control with G-box retained its fluctuation in both LD and DD. We further transformed *A. thaliana* with the luciferase gene (*LUC*) driven by an 1400 bp fragment upstream *QH6* with its G-box intact or mutated, respectively. The luciferase activity assay showed that a peak in the early morning disappeared in the mutant. Gene expression analysis also demonstrated that the rhythmic expression of *LUC* was abolished in the *hy5-1* mutant.

## Introduction

As sessile organisms, higher plants are capable of anticipating daily and annual requirements to coordinate biochemical and physiological activities. This helps plants to optimize their growth and adaptation and may confer selective advantages (Green et al., 2002; Dodd et al., 2005). The circadian clock is the key machinery regulating plant metabolism and development (Kreps and Kay, 1997) and can be modulated by external light and temperature signals, which also fluctuate daily and annually (McClung, 2001).

Kloppstech (1985) showed that mRNA levels of genes encoding light-harvesting chlorophyll a/b protein, Rubisco small subunit protein, and early light-inducible protein (ELIP) varied according to diurnal and circadian rhythms. This was probably the earliest report on plant circadian gene expression. Now it has been estimated that approximately one-third of *Arabidopsis thaliana* genes are regulated by the circadian clock at the transcriptional level (Michael and McClung, 2003; Edwards et al., 2006; Michael et al., 2008), not only for photosynthesis-related processes but also for functions such as metabolic adaptation, hormone signaling and photomorphogenesis (Harmer et al., 2000; Covington and Harmer, 2007).

In addition to photosynthesis and other primary metabolic activities, plant secondary metabolism has also been studied on a long-term basis. The biosynthesis and emission of volatile organic compounds (VOCs), including floral scents and green leaf volatiles, have also drawn much attention because of their economic and ecological impacts. Many plants were found to release VOCs from their leaves and flowers, and specific rhythmic emissions were reported in *Nicotiana* spp. (Raguso et al., 2003), *Quercus robur* (Bruggemann and Schnitzler, 2002), *Hoya carnosa* (Altenburger and Matile, 1990) and a number of other plants, including *A. thaliana* (Chen et al., 2003). Hemiterpene (isoprene) and monoterpenes (e.g., linalool and pinene) are major constituents of VOCs and possess important physiological and/or chemical ecological functions. It has been clearly demonstrated that the emission of isoprene and monoterpenes could protect photosynthetic organelles from photooxidative damage and increase their tolerance to heat (Sharkey and Yeh, 2001; Peñuelas and Llusià 2002, Peñuels, 2005). Plants can also regulate their flower scent by emitting a distinctive banquet of volatiles at different light phases as an adaptation for pollinators, which are active either in the day or at night (Kolosova et al., 2001; Raguso et al., 2003).

Although there is a large body of evidence showing that both biosynthesis and emission of terpenoid volatiles can be circadian-regulated, the molecular regulatory mechanisms are not well understood. In 2002, a β-pinene synthase gene (*QH6*) from *Artemisia annua* was isolated and found to be expressed in a circadian pattern at the transcriptional level (Lu et al., 2002). While terpene synthases were known to directly account for terpene formation, further studies showed that the steady-state abundances of transcripts of certain enzymes in upstream pathways and at metabolic branching points also fluctuated diurnally (Cordoba et al., 2009). This suggested that specific transcription factors might be involved in the global regulation of terpene metabolism at the pathway level.

In terms of the molecular machinery involved in plant terpenoid metabolic regulation, phytochromes and cryptochromes were found to be involved in the crosstalk between mevalonate (MVA) and methylerythritol phosphate (MEP) pathways, both of which provide the common substrate isopentenyl pyrophosphate (IPP) for terpenoid biosynthesis (Lichtenthaler, 1999). It was also suggested that HY5 (LONG HYPOCOTYL5), a bZIP transcription factor, might integrate signals from photoreceptors to suppress MVA pathway gene expression after illumination (Rodriguez-Concepción et al., 2004). Lee et al. (2007) did a genome-wide survey to identify *A. thaliana* HY5 binding sites by chromatin-immunoprecipitation using DNA chip hybridization (ChIP-chip), and revealed that at least a 2*C*-methyl-D-erythritol 2,4-cyclodiphosphate synthase (At1g63970) in the upstream MEP pathway, two terpene synthases (At3g14520, At3g25830), and one geranylgeranyl pyrophosphate synthase (GGPS, At4g38460) are light induced and contain HY5 binding sites in the promoter region of their genes. Zhang et al. (2011) also showed that HY5 could bind to over 9000 genes, of which more than 1100 genes were detectably affected in their expression.

In the present work, we isolated the upstream promoter sequence of the circadian-expressed β-pinene synthase gene *QH6* from *A. annua*, which we reported previously (Lu et al., 2002). An HY5 binding motif, G-box, was located and found to modulate gene expression. To the best of our knowledge, this is the first report on the regulation of monoterpene biosynthesis by transcription factor binding.

## Materials and methods

### Plant materials and growth conditions

Seeds of *A. annua* were kindly provided by Dr. Xiao-Ya Chen at Shanghai Institutes for Biological Sciences. Seeds were surface-sterilized and germinated in flasks on 0.3% (w/v) Gelrite (Duchefa Biochemie B.V., Haarlem, The Netherlands) with 1/2 Murashige and Skoog (MS) medium (Murashige and Skoog, 1962). Seedlings that were approximately 10 cm in height were moved into the soil. Seeds of *A. thaliana* Col-0, Ler-0 and *hy5-1* (Ler-0 background) lines from ABRC (Arabidopsis Biological Resource Center) were stratified at 4°C in dark for 2 days and then grown in pots containing a mixture of peat and vermiculite (3:1, v/v). Growth conditions for both *A. annua* and *A. thaliana* were 12h/12h light/dark photoperiod at 22°C, with a light intensity of 100 μmol m^−2^ s^−1^.

### Isolation of genomic DNA and total RNA, and reverse transcription

Genomic DNA was extracted from *A. thaliana* and *A. annua* following the standard CTAB protocol (Porebski et al., 1997). Total RNA was isolated from leaves using TRIzol reagent (Invitrogen) following the manufacturer's instructions. One microgram of total RNA was reverse-transcribed using the PrimeScript 1st Strand cDNA Synthesis Kit (TaKaRa) with oligo dT primer in a 20-μL system following the manufacturer's instructions. cDNA was stored at −80°C till further use.

### Cloning of the *QH6* gene and its upstream flanking sequence

The full-length cDNA sequence of *QH6* (GenBank Accession No. AF276072.1) (Lu et al., 2002) was aligned with both genomic and cDNA sequences of a myrcene/(*E*)- β-ocimene synthase gene of *A. thaliana* (At4g16740) (Bohlmann et al., 2000) to estimate the positions of the exons and introns (Supplementary Figure S1). Because there was no information on the gene size of *QH6*, to increase the accuracy and efficiency of amplification, we designed primers according to the alignment to amplify *QH6* genomic DNA in three overlapping fragments (primers and sequences are listed in Supplementary Table S1), each of which covers two predicted introns. Sequences of three amplified fragments were assembled. Approximately 0.1 μg genomic DNA of *A. annua* was amplified again with primers targeting two ends of the cDNA sequence (H-F and E-R) to confirm the assembly. Polymerase chain reaction (PCR) amplifications in this research were all conducted using LA Taq (TaKaRa) following the manufacturer's instructions, unless specifically noted. All amplicons were purified after agarose gel electrophoresis and subcloned into pGEM-T (Promega) to transform *Escherichia coli* DH5α and were sequenced for confirmation following standard molecular operation protocols (Sambrook et al., 1989). Sequences of all oligonucleotides used in this study are listed in Supplementary Table S1.

**Figure 1:**
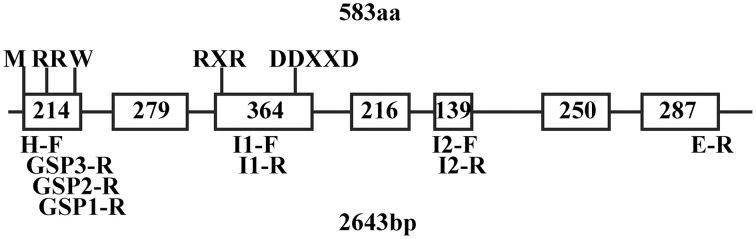
***QH6* gene structure showing sites and sizes of exons and introns, predicted conserved domains, and positions of designed primers**. M, methionine as a translation start signal; RR(X)_8_W, conserved tandem arginine as a transit peptide signal (R^48^R^49^), and a conserved tryptone (W^58^) at the 9th residue after RR. The conserved RXR domain (R^300^D^301^R^302^) used to direct the diphosphate anion away from the reactive carbocation after ionization and the DDXXD (D^337^D^338^V^339^Y^340^D^341^) domain for divalent cation (typically Mg^2+^ or Mn^2+^)-assisted substrate binding were both found at the 4th exon. Sequences of primers are listed in Supplementary Table S1.

The upstream flanking sequence of *QH*6 was cloned using the genome walking strategy, following the instruction of a GenomeWalker Universal Kit (Clontech). Approximately 160 μg genomic DNA of *A. annua* was digested in 100 μL with 80 units of *Dra*I, *Eco*RV, *Pvu*II, and *Stu*I (Promega) for 15 h and then ligated to adaptors at 16°C overnight. Three gene-specific reverse primers (GSP1-R, GSP2-R, and GSP3-R, Figure 1) were designed from the first extron of *QH6* to amplify the upstream fragment with adaptor primers AP1 and AP2 by nested PCR.

### Sequence analysis

Approximately 20 μg genomic DNA of *A. annua* was digested in a 50 μL system with *Cla*I, *Hin*dIII, and *Apa*LI overnight, respectively, resolved in a 1.2% agarose gel and blotted onto a Hybond N+ membrane (GE Healthcare). A 442-bp gene-specific fragment was amplified by primers Probe-F and Probe-R (Supplementary Table S1) and purified as a template for probe preparation. AlkPhos Direct Labeling Reagents and a CDP-Star™ Kit (GE Healthcare) were used for labeling and detection following the manufacturer's instructions. Membranes with probes were exposed to Kodak X-Omat Blue film (Perkin Elmer, Waltham, MA) for 48 h. The film was developed using standard methods.

We searched the region upstream from the *QH6* translation initiation site for known *cis*-elements in plants using PLACE (A Database of Plant *Cis*-acting Regulatory DNA Elements) (Higo et al., 1999) and NSITE-PL (RegSite Plant DB, Softberry Inc., Mount Kisco, NY) databases.

### Binary constructs and plant transformation

Different truncations of the *QH6* promoter region were amplified from the cloned upstream fragment using a common reverse primer, QH6-R, of which two nucleotides were mutated to generate an *Nco*I site for subsequent gene expression, and different forward primers with a *Pst*I restriction site. All amplified promoter fragments were purified after gel electrophoresis, subcloned into the pGEM-T vector, and confirmed by sequencing.

**Figure 2:**
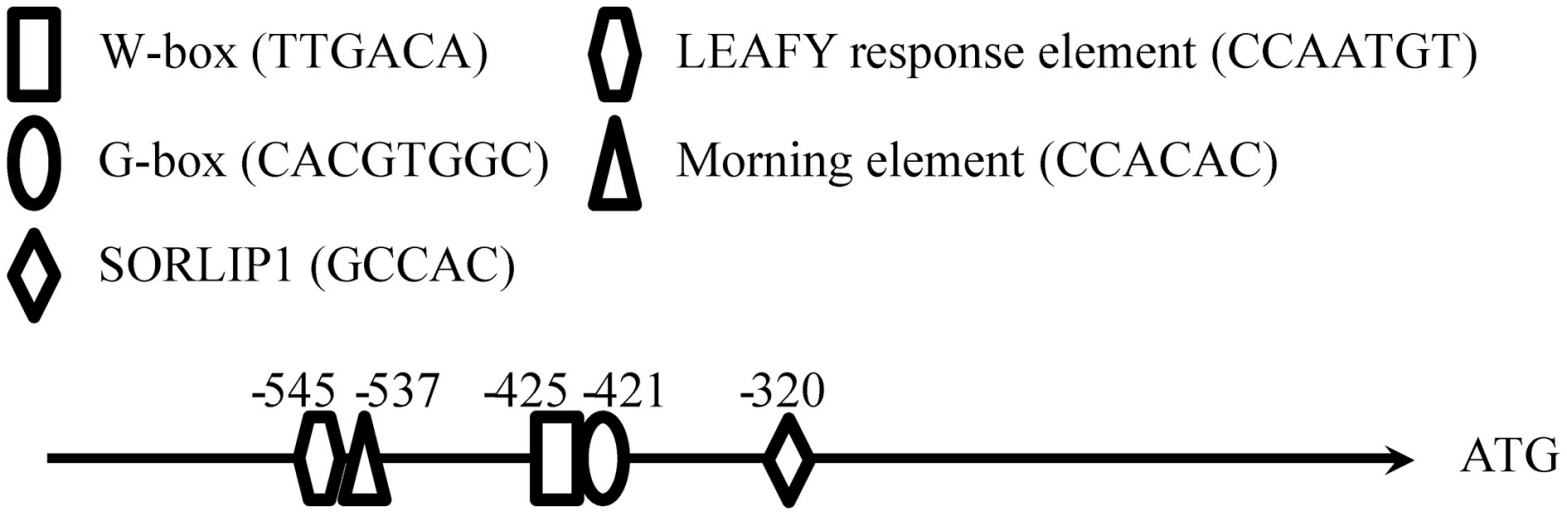
**Sequence analysis of the *QH6* promoter and the construction of different promoter truncations driving *GUS* expression in *Arabidopsis thaliana***. The sequence from −800 bp to the *QH6* translation initiation site was searched for known regulatory elements in PLACE and NSITE-PL databases to identify any known motifs, as described in Materials and Methods Section.

For expressing *GUS* in *A. thaliana*, two different truncations immediately before and after G-box of *QH6* promoter were amplified using forward primers QH6+GB-F and QH6-GB-F, respectively, with QH6-R. Each of the forward primers contain a *Pst*I site at the 5′ end for further cloning work. Both of the promoter truncations were digested by *Nco*I and *Pst*I and ligated to the same digested pCAMBIA1391Z vector (CAMBIA) to drive *GUS* expression. These constructs were named GB+ (with promoter beginning at the predicted G-box), and GB− (with promoter beginning immediately after the predicted G-box) (Figure 2).

For expressing luciferase gene (*LUC*), the 1400 bp fragment upstream translation initiation site was amplified using QH6-1400-F, which also contains a *Pst*I site at the 5′-end, and QH6-R. To mutate its G-box core from CACGTG to ACGATC, a megaprimer strategy was adopted (Ke and Madison, 1997). In brief, the forward primer QH6-1400-MF was used with QH6-R to amplify the first short fragment and to introduce the mutation. This fragment was purified and used as a megaprimer with 1400-F to generate the 1400 bp promoter fragment with the G-box changed. Full open reading frame of luciferase gene (*LUC*) was digested from pSPLuc+NF (Promega) and then cloned between *Nco*I and *Hpa*I sites in pCAMBIA1390 (CAMBIA) to give pCAMBIA1390-LUC. 1400 (1400 bp full length promoter of *QH6*) and 1400M (mutate G-box of 1400) were fused between *Pst*I and *Nco*I in pCAMBIA1390-LUC to give 1400::LUC and 1400M::LUC, respectively.

Constructs were transformed into *Agrobacterium tumefaciens* GV3130 by electroporation with an Eppendorf 2510 electroporator (Yi and Kao, 2008). A positive colony for each construct was confirmed by PCR and inoculated for *A. thaliana* transformation by floral dipping according to Clough and Bent (Clough and Bent, 1998).

T0 seeds were germinated on 0.3% Gelrite plates with 1/2 MS medium (Murashige and Skoog, 1962) and 15 μg/mL hygromycin for screening. Seedlings growing well were moved into pots containing a mixture of peat and vermiculite (3:1, v/v) after 2 weeks. Genomic DNA was extracted from mature leaves to study the copy number and T-DNA insertion site by genome walking. Ten independent homozygote T2 lines were screened by PCR according to the T-DNA Primer Design Tool (http://signal.salk.edu/tdnaprimers.2.html) and the previous genome walking result (data now shown).

### Gene expression analysis

For *A. thaliana* plants transformed with different promoter truncations, mature leaves from homozygous transgenic plants with approximately 12 rosette leaves were sampled every 2 h for two continuous days and were frozen in liquid nitrogen immediately. RNA was isolated from a bulk of leaves from at least four plants to minimize individual variation, and 1 μg was reverse-transcribed as described above. To quantify gene expression, a 25-μL reaction system contained 12.5 μL SYBR Premix Ex Taq II (TaKaRa), 1 μL of each forward and reverse primers, 0.4 μL cDNA template and 9.1 μL RNase-free H_2_O. A Thermal Cycler Dice Real Time System TP800 (TaKaRa) was used and the reaction program was 95°C for 30 s, followed by 40 cycles of 95°C for 5 s and 60°C for 30 s.

The second exon of *GUS* was amplified by primers GUS-Q-F and GUS-Q-R for real-time quantification. *Actin8* (Hewezi et al., 2010) was amplified by primers ACT8-Q-F and ACT8-Q-R as a reference for normalizing *GUS* expression.

For each sample, PCR assays were carried out in triplicate. Melting curve analysis was used to confirm the specificity of the method. The results were analyzed using a TP800 Thermal Cycler Dice Real Time System (Takara). The threshold cycle (CT) values were automatically determined by the TP800 software (Takara). Expression values were calculated according to the 2^−ΔCT^ method (for each gene, ΔC_T_ = C_T,Gene_ − C_T,actin_) (Livak and Schmittgen, 2001).

For luciferase activity assay, the luminescent signal was detected and 4 min exposure images were acquired by IVIS Lumina XR Imaging system (Perkin Elmer). 2.5 mmol/L luciferin (with 0.01% Triton X-100) was sprayed 1 h before detection (Millar et al., 1992). The radiance was calculated by Living Image Software and ROI (Region of Interest) tool.

In all treatments, the time when lights were switched on (dawn) was defined as Zeitgeber time 0 (ZT0). Prism (GraphPad Software) was used to perform the statistic analysis. Results were presented as mean ± SD. Transcript abundances were expressed as relative values to corresponding ZT0 levels of each line. An unpaired Student's *t*-test was conducted to examine the differences between the groups.

### Yeast one hybrid assay and electrophoretic mobility shift assay

The fragment from −258 to −425 bp upstream of the *QH6* translation initiation site was amplified by PCR with primers +GB-F and GB-R and was subcloned into the *Eco*RI/*Spe*I sites of pHIS2.1 (Clontech) as +GB. G-box -deleted (-GB) and -mutated (GBM) fragments were amplified using reverse primer GBR and forward primer -GB-F or GBM-F, respectively, and were fused to pHIS2.1. A fragment containing four tandem repeats of G-box (GACACGTGGC) was synthesized by annealing 4GB-F and 4GB-R primers (Supplementary Table S1) and cloned to pHIS2.1 as 4GB. The HY5-AD fusion vector was constructed by amplifying the *HY5* (At5g11260) open reading frame from *Arabidopsis* cDNA with primers HY5-F and HY5-R and introducing the purified PCR product to the *Sma*I site of pGADT7-Rec2 (Clontech). The plasmids were transformed into *Saccharomyces cerevisiae* Y187 and HY5/+GB, HY5/-GB, HY5/GBM and HY5/4GB double transformants were selected on -Trp/-Leu double drop-out plates. A binding ability assay was performed by dotting 5 μL liquid culture on -Trp/-Leu/-His triple drop-out plates. The liquid culture was series-diluted while the initial concentration of OD_600_ = 0.1 was taken as 0.1.

For EMSA, the *HY5* open reading frame was amplified with primers HY5EX-F and HY5EX-R from *A. thaliana* cDNA and cloned into pGEX-4T-1 (GE Healthcare) between *Eco*RI/*Xho*I sites. The expression construct was transformed into *E. coli* BL21(DE3)pLysS (Invitrogen) for prokaryotic expression. The GST-HY5 fusion protein was purified using a MagneGST protein purification system (Promega). The biotin labeled 52-bp probes of the *QH6* promoter containing G-box (391-442) and the mutant (M391-442) were synthesized by GenScript (Nanjing). An unlabeled probe was used as a competitor. The GST-only protein was purified and was used as a negative control. The interaction of the HY5 protein and DNA fragment was determined using a LightShift Chemiluninescent EMSA kit (Pierce) following the manufacturer's instruction. The 20-μL binding system was resolved on an 8% polyacrylamide gel in 1 × TBE and was semi-dry blotted onto Hybond-N+ membrane (GE Healthcare). After a three-hour cross-linking at 60°C, the signal was detected according to the manufacturer's instruction with Kodak X-Omat Blue film (Perkin Elmer, Waltham, MA).

## Results

### *QH6* sequence analysis

According to the alignment of the *A. annua QH6* cDNA sequence (Lu et al., 2002) with *A. thaliana* ocimene/myrcene synthase (At4g16740) cDNA and genomic DNA sequences (Chen et al., 2003), approximate positions for introns and exons of *QH6* were predicted (Supplementary Figure S1). Three primer pairs were designed to amplify *QH6* genomic DNA into three overlapping fragments, each of which covered two introns according to the prediction. The amplified fragments were assembled and re-confirmed by amplifying *A. annua* genomic DNA with primers H-F and E-R from two ends of the *QH6* coding region. The *QH6* gene has seven exons and six introns with the highly conserved tandem arginine domain (RR) in the first exon and the aspartate-rich divalent binding domain (DDXXD) in the fourth exon, as a typical Class III terpene synthase (Trapp and Croteau, 2001; Aubourg et al., 2002; Lange and Ghassemian, 2003). There was no hit with obvious similarity when we searched GenBank with sequence of any of introns (data not shown). The gene structure and positions of conserved domains and designed primers are shown in Figure 1.

The sequence upstream of *QH6* was cloned by genome walking, and the first 2-kb fragment flanking the *QH6* translation initiation site was confirmed by re-amplification from *A. annua* genomic DNA directly. By searching databases of PLACE (A Database of Plant *Cis*-acting Regulatory DNA Elements) (Higo et al., 1999) and NSITE-PL (RegSite Plant DB, Softberry Inc., Mount Kisco, NY), an HY5-binding G-box motif (CACGTGGCA) was found at −421 bp (Figure 2). Other motifs, such as SORLIP1 (GCCAC, −320), LEAFY response element (CCAATG, −545), W-box (TTGAC core, −425), and Morning element (ME, CCACAC, −537) were also found (Priest et al., 2009) (Figure 2). The entire *A. annua QH6* sequence, including the upstream promoter region, was deposited in GenBank under the accession number GU929215.

When *QH6* was originally isolated and characterized, there was no information suggesting that it was a single copy gene (Lu et al., 2002). This raised the possibility that other genes with a high sequence identity to *QH6* might be analyzed, either instead of or together with *QH6* itself. Here, by Southern blotting, a single band was detected from *Cla*I-, *Hin*dIII-, and *Apa*LI-digested genome DNA samples (2471, 2269, 1267 bp), confirming that the *A. annua* genome has only one copy of *QH6* and providing a rationale for our subsequent studies (data not shown).

### Gene expression driven by *QH6* promoter truncations

To figure out whether the G-box functions in regulating *QH6* expression, we expressed β-glucuronidase gene (*GUS*) driven by the *QH6* upstream fragment immediately before (GB+) or after G-box (GB−), respectively, in *A. thaliana*. For each construct, 10 independent transgenic lines were screened and genome walking was adopted to study the insertion site of each line to ensure that T-DNA insertion did not interrupt any gene known or presumed to affect either *GUS* expression or terpenoid metabolism (data not shown).

**Figure 3:**
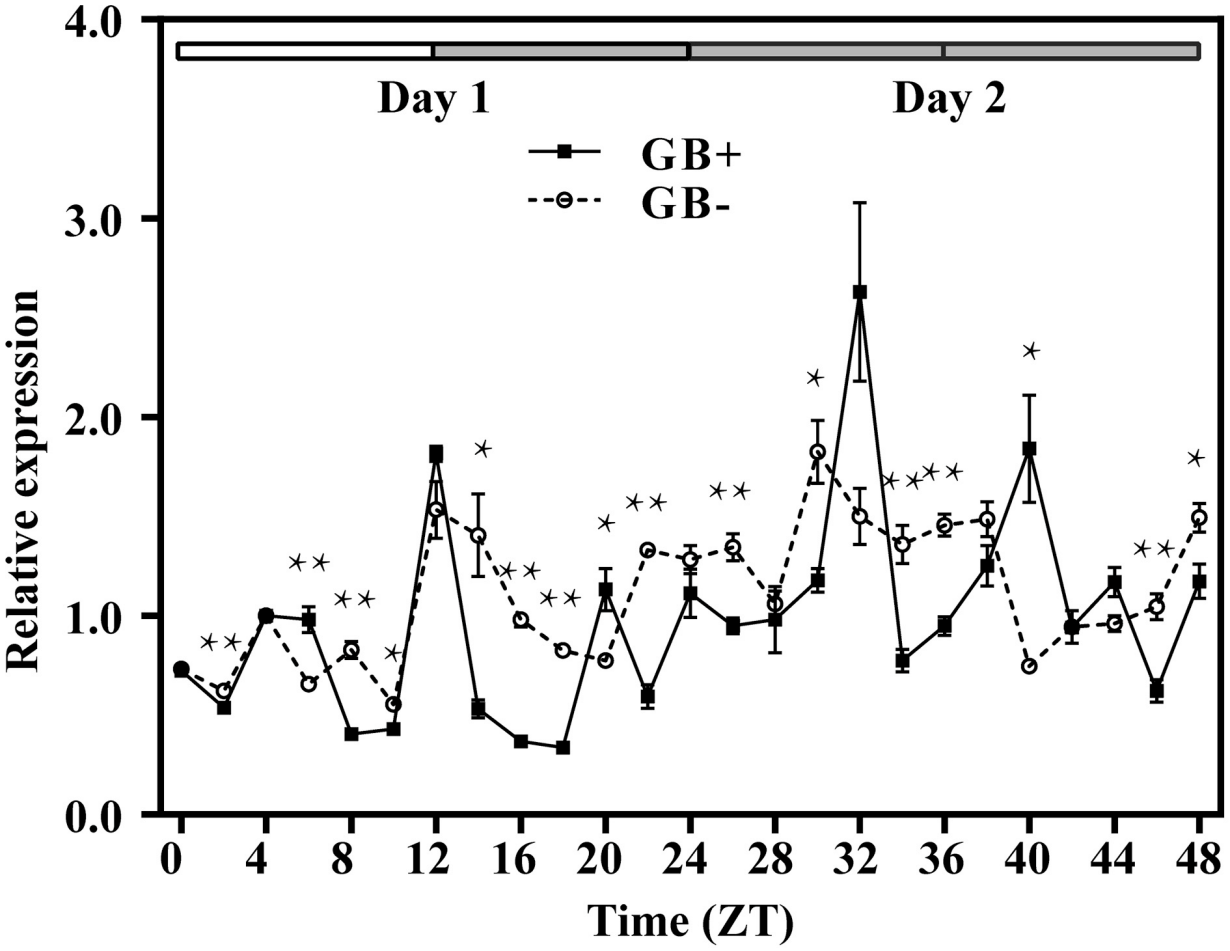
**Rhythmic expression of *GUS* driven by the upstream fragment of the *QH6* promoter immediately before (GB+) or after (GB−) the G-box motif**. *Arabidopsis thaliana* plants were transformed by GB+ or GB− constructs. The steady-state level of *GUS* transcripts was quantified by real-time PCR, using *Actin8* as a reference gene. Leaves were sampled in triplicate for two continuous days, with the first day in standard 12h/12h light/dark cycle and the second day in constant dark. ZT is Zeitgeber time and here represents time from light on at dawn. Filled and open bars indicate subjective days and nights. Data represent means ± SD. The differences of expression levels were assessed by unpaired Student's *t*-test. ^*^p < 0.05 and ^**^p < 0.01 in GB+ vs. GB− transgenic Col-0 plants.

For both GB+ and GB− plants, rosette leaves were sampled every 2 h for 1 day in 12h/12h light/dark (LD) condition (Figure 3, from ZT0 to ZT24) and then for 24 h in constant dark (DD) (Figure 3, from ZT24 to ZT48). In GB+ plants in LD, two peaks of the steady state *GUS* transcripts were detected at ZT08 and ZT16, corresponding to the afternoon and midnight in nature. In DD, the fluctuation of *GUS* transcript abundance also showed two peaks at ZT28 and ZT36, both were 4 h ahead their LD counterparts. The ratios of transcript abundances at the highest level to the lowest were 5.39 in LD and 4.42 in DD, showing a significant fluctuation. For GB− plants, we could not identify a clear rhythmic change as the fluctuation of transcript abundance looked more frequently and the highest-to-lowest ratios were only 2.77 and 2.35 in LD and DD, respectively (Figure 3).

**Figure 4:**
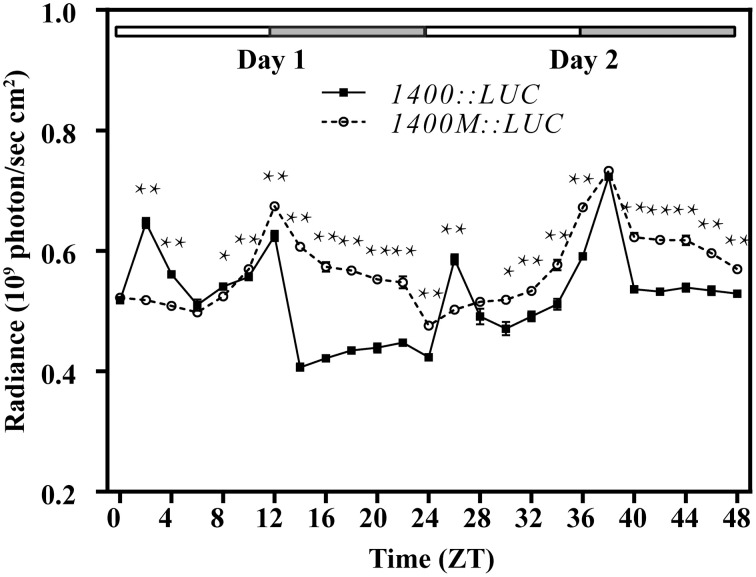
**Diurnal expression of the luciferase gene (*LUC*) driven by the 1400 bp upstream fragment of the *QH6* promoter, with the G-box intacted (1400::LUC) or mutated (1400M::LUC)**. *Arabidopsis thaliana* Col-0 plants were transformed by 1400::LUC and 1400M::LUC constructs. The activity of luciferase expressed was quantified by measuring the radiance emitted and captured in 4 min by IVIS Lumina XR Imaging System (Caliper Life Sciences). Plants were growing in standard 12h/12h light/dark conditions. ZT is Zeitgeber time and here represents time from light on at dawn. Filled and open bars indicate subjective days and nights. Data represent means ± SD. The differences of expression levels were assessed by unpaired Student's *t*-test. ^*^p < 0.05 and ^**^p < 0.01 in 1400::LUC vs. 1400M::LUC transgenic Col-0 plants.

For better understanding the function of this G-box, we transformed *Arabidopsis* with luciferase gene (*LUC*) driven by an 1400 bp promoter fragment of *QH6* with its G-box either intact or mutated from CACGTG to ACGATC to abolish its possible function. Transformed lines were screened and confirmed by genome walking as mentioned above. We monitored the luciferase activity by measuring radiance emitted with an IVIS Lumina XR Imaging System (Caliper Life Sciences) every 2 h under LD condition for 2 days. For control lines transformed with intact G-box (1400::LUC), we observed two peaks at ZT02 and ZT12 in Day 1, and another two peaks at ZT26 and ZT38 in Day 2. However, for the mutant lines (1400M::LUC), the ZT02 and ZT26 peaks no longer existed whereas the ZT12 and ZT38 peaks stayed as the control (Figure 4).

**Figure 5:**
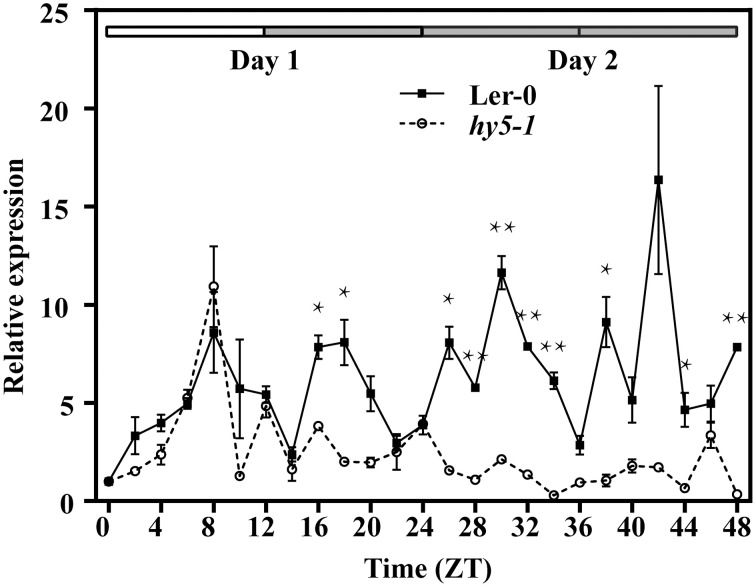
**Rhythmic expression of the luciferase gene (*LUC*) driven by the 1400 bp upstream fragment of the *QH6* promoter in transgenic *Arabidopsis thaliana* Ler-0 and *hy5-1* mutant lines**. The steady-state level of *LUC* transcripts was quantified by real-time PCR, using *Actin8* as a reference gene. Leaves were sampled in triplicate for two continuous days, with the first day in standard 12h/12h light/dark cycle and the second day in constant dark. ZT is Zeitgeber time and here represents time from light on at dawn. Filled and open bars indicate subjective days and nights. Data represent means ± SD. The differences of expression levels were assessed by unpaired Student's *t*-test. ^*^p < 0.05 and ^**^p < 0.01 in 1400::LUC transgenic Ler-0 vs. 1400::LUC transgenic *hy5-1*.

We then transformed Ler-0 and *hy5-1* with 1400::LUC to study the expression of *LUC* when *HY5* was silenced. The transcript abundance of *LUC* showed a synchronized accumulation in both lines under normal light/dark photoperiod (Figure 5). However, when entrained plants were placed into constant dark, only in 1400::LUC-transformed Ler-0 plants could a rhythmic fluctuation in *LUC* expression level be identified (Figure 5). The abundance of *LUC* transcripts in the transformed *hy5-1* mutant decreased to a much lower level, and its fluctuation was not as significant as what we observed in Ler-0 (Figure 5).

### Yeast one-hybrid and electrophoretic mobility shift assay (EMSA)

**Figure 6:**
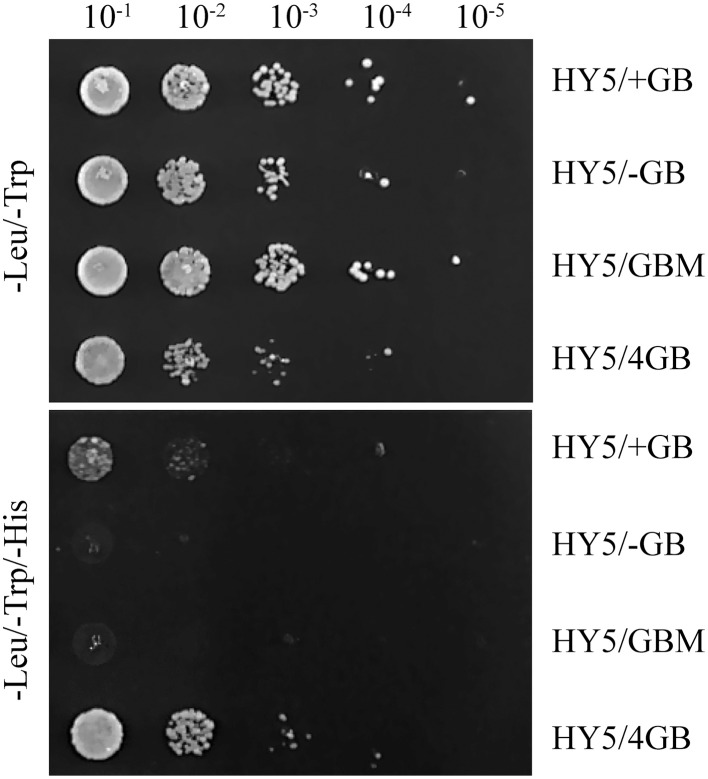
**The yeast one-hybrid showed that the *QH6* promoter region could bind HY5 *in vivo***. The *QH6* promoter fragment from −258 to −425 bp upstream of the translation initiation site, including the G-box, was used as the bait (+GB), and the same fragment with the G-box removed (-GB) or mutated (GBM) was used as the control. A sequence containing four consecutive repeats of the G-box motif (4 GB) was also synthesized to test enhanced interactions. pHIS2.1 harboring +GB, -GB, GBM, or 4GB was co-transformed with pGADT7-HY5. Cells were grown in liquid medium to OD_600_ = 0.1 (10^−1^) and diluted in a 10 × series (10^−2^ to 10^−5^). At each dilution, 5 μ L was spotted on media selecting for the existence of both plasmids (-Leu/-Trp double drop-out) or the interaction (-Leu/-Trp/-His triple drop-out).

To test whether the *QH6* promoter binds HY5, a fragment from −258 to −425 bp to the *QH6* translation initiation site was amplified by polymerase chain reaction (PCR) as a bait (+GB) for binding assays by yeast one-hybrid. The interaction between HY5 and this promoter fragment was screened by growth on -Trp/-Leu/-His triple dropout plates. Different concentrations of 3-aminotriazole (3AT), a histidine synthase inhibitor, were supplemented to the media to suppress background activation. These assays showed that the −258 to −425 bp region of the *QH6* promoter was able to bind HY5 *in vivo* (Figure 6). The assay with a bait containing four consecutive repeats of the G-box motif showed strong interaction with HY5, whereas that with G-box either removed or mutated had no interaction.

**Figure 7:**
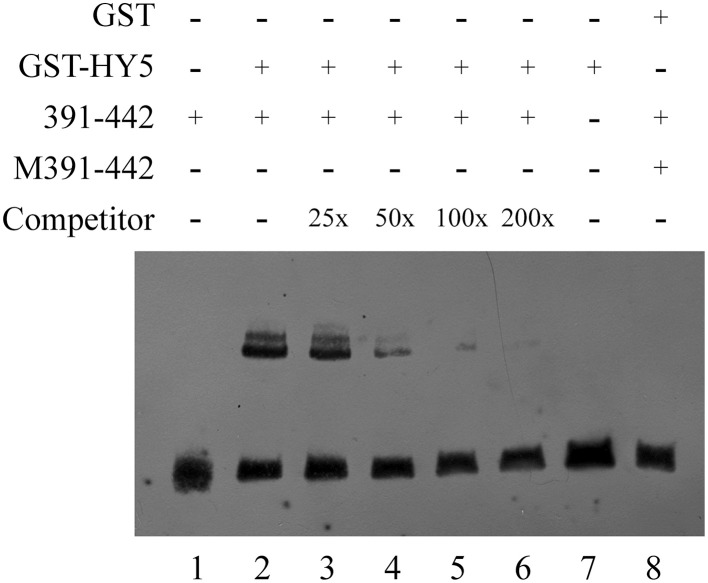
**EMSA confirmed the interaction between HY5 and the *QH6* G-box motif *in vitro***. GST and GST-HY5 proteins were expressed and purified as described in the Materials and Methods Section. For each binding reaction, 5 μ g of recombinant protein was used. A double-stranded probe was generated by annealing synthesized single strand primers. The −391 to −442 bp fragment of the *QH6* promoter was biotin-labeled as 391-442, while M391-442 represents a mutant probe. In each lane, 20 fmol of biotin-labeled probe was used. In lane 1, only 20 fmol of the wild-type probe was used, while in lane 2, 20 fmol of the wild-type probe was used along with 5 μ g GST-HY5 protein. An unlabeled probe was used as a competitor as marked on the left. In lanes 3–6, unlabeled competitors (0.5 pmol, 1 pmol, 2 pmol, 4 pmol, respectively) were added to the binding assay with the GST-HY5 protein and the 391-442 biotin-labeled probe. The relative concentration ratio was marked as in the competitor row. Negative controls were 20 fmol mutant probe together with 5 μ g GST-HY5 protein (Lane 7) and 20 fmol wild-type probe with 5 μ g GST protein (Lane 8).

EMSA was performed to further determine the binding of HY5 to a specific DNA motif in this promoter region. A biotin-labeled 52-bp double-strand DNA probe containing the G-box motif and its flanking region was synthesized, while HY5 was prokaryotically expressed and purified as described in the Materials and Methods Section. The labeled probe was incubated with or without HY5 protein preparation and separated on native polyacrylamide gels. Shifted bands were routinely observed with HY5 and *QH6* G-box oligonucleotides that were abolished by using a competitor against the entire 52-bp region. Thus, we confirmed that HY5 binds to the *QH6* promoter at the G-box motif (Figure 7).

## Discussion

The *A. annua* β-pinene synthase gene *QH6* is among a small number of terpene synthase genes reported to be regulated by the circadian clock at the transcriptional level. Its expression was found to be suppressed by either mechanical wounding or fungal elicitor (Lu et al., 2002). However, two other *A. annua* monoterpene synthase genes (*QH1* and *QH5*), both of which encode linalool synthases, were induced by mechanical wounding (Jia et al., 1999), and a sesquiterpene (caryophyllene) synthase gene *QHS1* was capable of being upregulated by fungal elicitor (Cai et al., 2002). This suggested that the plant could fine-tune its metabolic flux among different terpenoid metabolic branches in response to environmental stimuli. Although monoterpene synthases all function spatially in plastids, diurnal expression in higher plants might provide the potential to temporally separate these enzyme activities for better adaptation.

For terpene metabolism, not only monoterpene synthases but also enzymes for hemiterpene, sesquiterpenes and carotenoid biosynthesis showed rhythmic fluctuations at the transcriptional level. For example, both isoprene (hemiterpene) synthase and phytoene synthase (for carotenoid biosynthesis) in the poplar displayed a diurnal variation in gene expression (Loivamäki et al., 2007). Similarly, a (*E*)-β-caryophyllene (sesquiterpene) synthase gene (*OsTPS3*) from rice (*Oryza sativa* L.) showed circadian regulation, with its peak transcript abundance appearing in the morning (Cheng et al., 2007). However, we are still far from achieving a molecular understanding of the endogenous rhythmicity of plant terpene metabolism.

The bZIP transcription factor HY5 (LONG HYPOCOTYL5) has been found to bind G-box and to promote activation of a number of light-induced genes, such as Rubisco small subunit (Ang et al., 1998; Chattopadhyay et al., 1998). There are also results showing that the G-box was over-represented in eight of nine promoters for rhythmically co-expressed genes in flavonol/anthocyanin metabolic pathway, suggesting that HY5 plays an essential role in plant secondary metabolism regulation through the G-box (Pan et al., 2009). In our work, *GUS* driven by *QH6* promoter truncation immediately after G-box didn't reveal a significant rhythmic fluctuation as we reported in *A. annua* before (Lu et al., 2002). This infers that G-box motif is essential for regulating this monoterpene synthase gene expression. Both yeast one-hybrid and EMSA studies in our work confirmed that this regulation is through the direct binding by the bHLH transcription factor HY5. Moreover, when we compared the expression of *LUC* under the control of the promoter region of *QH6* in Ler-0 and *hy5-1*, the rhythmic fluctuation of the steady state abundance of *LUC* transcripts turned to be insignificant in *HY5*-silencing plants. This suggests that HY5 is essential for the rhythmic expression of *QH6*.

Because the transcript abundance of *GUS* and the enzyme activity of luciferase reached their highest levels at different time phases under the control of two different native promoter truncations of 1400 bp (1400::LUC) and 424 bp (GB+), it is possible that other regulatory elements upstream G-box exist between −1400 and −424. When we searched for known *cis*-elements, a morning element (ME) motif CCACAC was found at −537. It has been reported that ME and G-box might construct a “phase module” (Michael et al., 2008), with the core specifies the time of the day when the gene is expressed and the flanking sequence refines the exact phase. In our transgenic *Arabidopsis* plants, luciferase activity fluctuated differently between 1400::LUC and 1400M::LUC lines, of which only the G-box core motif regions were different (Figure 4). This suggested an interaction between G-box and ME, and supported that the expression of the monoterpene synthase gene *QH6* was probably finely tuned by G-box and ME. However, it is currently unclear how multiple factors are coordinated *in planta* for *QH6*.

In addition to this, it is worthwhile to note that, there are two other putative elements around G-box and ME. A LEAFY response element (CCAATGT) locates only two nucleotides upstream ME, and an atypical W-box (TTGACA) overlaps with G-box. It is yet to be determined if these two elements confer any further regulation on *QH6* gene expression in response to developmental or resistance signal, respectively (Maleck et al., 2000; Kamiya et al., 2003).

To the best of our knowledge, this was the first report of HY5 regulation of plant gene expression for monoterpene biosynthesis. Our results suggested a short signaling pathway from HY5 to plant secondary metabolism by direct binding to the promoter, which modulates gene transcription. However, given the global regulatory function of HY5, it is also likely that induction of other genes/transcription factors by HY5 could be involved. Detailed studies on circadian rhythm, light induction, and the spatial/temporal specificity of *QH6* expression might help us to better understand the complicated regulatory network involved in plant terpene metabolism.

### Conflict of interest statement

The authors declare that the research was conducted in the absence of any commercial or financial relationships that could be construed as a potential conflict of interest.
