# Supplementary material for: The bZIP transcription factor HY5 interacts with the promoter of the monoterpene synthase gene QH6 in modulating its rhythmic expression
Source: Front Plant Sci. 2015 Apr 30;6:304. doi: 10.3389/fpls.2015.00304 (PMC4415419; doi:10.3389/fpls.2015.00304)
Supplement: Supplementary file 1 [file Table1.DOCX]

Supplement Table S1. Primers used in this study. Forward and reverse primers were named with “-F” and “-R” suffixes, respectively. Restriction sites were underlined.

| Primer | Sequence |
| --- | --- |
| *To amplify QH6 introns* | |
| H-F | 5'-ATGGCTTCAATGTGCACATTTTCTTCTCCA-3' |
| I1-F | 5'-CCACTTCATTGGATGGTTCCACGGG-3' |
| I2-F | 5'-CTTCCTTGCGTGCTACAATGCTACCAATG-3' |
| I1-R | 5'-CCCGTGGAACCATCCAATGAAGTGG-3' |
| I2-R | 5'-CATTGGTAGCATTGTAGCACGCAAGGAAG-3' |
| E-R | 5'-GGGTACCCCCTAGATTGGATTAACAAACAATG-3' |
| *To amplify 5’ upstream flanking region* | |
| AP1 | 5'-GTAATACGACTCACTATAGGGCACGCGTGGTCGACGGCCCGGGCTGGT-3' |
| AP2 | 5'-ACTATAGGGCACGCGTGGT-3’ |
| GSP1-R | 5'-GAAAGCGACTGGACAAAATCATAGGACCA-3' |
| GSP2-R | 5'-GGGCATAATTAGCTGATCTTCTGTTGGTGG-3' |
| GSP3-R | 5'-GGGCTTGTAGTGTAGATGTTTGCTTGTTACA-3' |
| *To amplify QH6 fragment for Southern Blotting analysis* | |
| Probe-F | 5'-GTTTGGTTGGTCCAAGTCGGGTTT-3' |
| Probe-R | 5'-CATCAGCCATTAGCTATGGAGCATCA-3' |
| *To amplify different truncates of QH6 upstream sequence for promoter analysis* | |
| QH6+GB-F | 5'-AACTGCAGTGACACGTGGCAAAATAATATCTATCTACCC-3' |
| QH6-GB-F | 5'-AACTGCAGAAATAATATCTATCTACCCACAACAAATC-3' |
| QH6-1400-F | 5’-AACTGCAGAATCACTCTG GAAATTTGAT CTAAATGATT AGAGGG-3’ |
| QH6-1400-MF | 5'-GCTCTATTTTGAACGATCGCAAAATAATATCTATCTACCC-3' |
| QH6-R | 5'-GTGCACATTGAAGCCATGGCTGCAAGGTGG-3' |
| *To quantify gene expression* | |
| GUS-Q-F | 5'-AGCCGATGTCACGCCGTATGTTAT-3' |
| GUS-Q-R | 5'-TGTAGAGCATTACGCTGCGATGGA-3' |
| LUC-Q-F | 5'-CAACTGCATAAGGCTATGAAGAGA-3' |
| LUC-Q-R | 5'- ATTTGTATTCAGCCCATATCGTTT-3' |
| ACT8-Q-F | 5'-TCACCACAACAGCAGAACGGGAAAT-3' |
| ACT8-Q-R | 5'-AAGGACTTCTGGGCACCTGAATCTC-3' |
| *For electrophoresis mobility shift assay* | |
| HY5EX-F | 5'-GGAATTCATGCAGGAACAAGCGACTAGCTCTTTAG-3' |
| HY5EX-R | 5'-CGCTCGAGTCAAAGGCTTGCATCAGCATTAGAACC-3' |
| B-391-442 | 5'-CCTTTAAGTTGCTCTATTTTGACACGTGGCAAAATAATATCTATCTACCCAC-3' |
| B-M391-442 | 5'-CCTTTAAGTTGCTCTATTTTGAACGATCGCAAAATAATATCTATCTACCCAC-3' |
| *For yeast-one hybrid* | |
| GB-F | 5'-GCGAATTCTGACACGTGGCAAAATAATATC-3' |
| GB-R | 5'-TTACTAGTCAGTAAGCATGTATTCAAGTTG-3' |
| -GB-F | 5'-GCGAATTCAAATAATATCTATCTACCA-3' |
| GBM-F | 5'-GCGAATTCTGAAAATAGCAAAATAATATC-3' |
| 4GB-F | 5'-AATTCGACACGTGGCGACACGTGGCGACACGTGGCGACACGTGGCA-3' |
| 4GB-R | 5'-CTAGTGCCACGTGTCGCCACGTGTCGCCACGTGTCGCCACGTGTCG-3' |
