# Supplementary material for: The bZIP transcription factor HY5 interacts with the promoter of the monoterpene synthase gene QH6 in modulating its rhythmic expression
Source: Front Plant Sci. 2015 Apr 30;6:304. doi: 10.3389/fpls.2015.00304 (PMC4415419; doi:10.3389/fpls.2015.00304)
Supplement: Supplementary file 2 [file Image1.PDF]

Supplement Figure 1. Alignment of the *QH6* cDNA sequence (GenBank Accession No. AF276072.1) and genomic (At4g16740g) and cDNA (At4g16740c) sequences of the *Arabidopsis thaliana* myrcene/(*E*)-β-ocimene synthase gene (At4g16740) to predict *QH6* splicing sites. The alignment was performed using Vector NTI (Invitrogen), and manually edited using GeneDoc (http://www.nrbsc.org/gfx/genedoc/). Identical nucleotides are shaded.

```
AF276072.1 : ------------------ATGGCTTCAATGTGCACATTTTCTTCTCCATTTCTCTTATGTAATAGTTCCATAAGTAGAAC
At4g16740c : --------------------------------------------------------------------------------
At4g16740g : AAATCAGTATAAAATATCATAGCGGAGTCTAAAGAAGAGTTCATCCAATTATTTAACTCTTTAGTGTAATCATTAGCAAC

AF276072.1 : AAATATTGTTGCATGTAACAAGCAAACATCTACACTACAAGCCCAAGTTAAAAATGTTGCTACAATTGAAACCACCAA--
At4g16740c : --------ATGCCTAAACGACAGGCTCAACGGCGTTTCACTCGCAAGACTGACTCGAAAACACCATCCCAGCCTCTGGTA
At4g16740g : GCAAAGAAATGCCTAAACGACAGGCTCAACGGCGTTTCACTCGCAAGACTGACTCGAAAACACCATCCCAGCCTCTGGTA

AF276072.1 : --CAGAAGATCAGCTAATTATGCCCCTTCATTATGGTCCTATGATTTTGTCCAGTCGCTTTCTAGCAAATACAAAGGAGA
At4g16740c : TCCCGTCGCTCTGCAAACTATCAACCGTCTCTTTGGCAGCACGAATATCTCCTCTCGCTCGGTAATACATATGTG-----
At4g16740g : TCCCGTCGCTCTGCAAACTATCAACCGTCTCTTTGGCAGCACGAATATCTCCTCTCGCTCGGTAATACATATGTGGTACG

AF276072.1 : TA------------------------------------------------------------------------------
At4g16740c : --------------------------------------------------------------------------------
At4g16740g : TATAAGAATGTACATGACGTTGTATTAAATAAAATTATGTGACATATATTACTATTTAGGAAATTATATATAGTGATAAT

AF276072.1 : -------------ACTATATGGCAAGATCACGAGCTCTAAAAGGAGTAGTGAGGACCATGATTTTAGAAGCGAATGGAAT
At4g16740c : ---------------AAAGAGGACAACGTCGAGAGAGTTACGTTATTGAAGCAGGAAGTGAGTAAAATGCTCAATGAAAC
At4g16740g : ATTTATATCATGCAGAAAGAGGACAACGTCGAGAGAGTTACGTTATTGAAGCAGGAAGTGAGTAAAATGCTCAATGAAAC

AF276072.1 : TGAAAATCCATTGAGTTTACTTAATTTGGTCGATGATTTGCAAAGACTTGGAATATCATATCATTTTTTGGATGAAATAA
At4g16740c : GGAAGGTTTACTCGAACAGCTAGAGCTCATCGACACTTTACAAAGGCTTGGAGTTTCTTACCATTTTGAACAAGAAATCA
At4g16740g : GGAAGGTTTACTCGAACAGCTAGAGCTCATCGACACTTTACAAAGGCTTGGAGTTTCTTACCATTTTGAACAAGAAATCA

AF276072.1 : GCAATGTTTTGGAGAAAATATACTTAAATTTCTACAAAAGTCCTGAAAAGTGGACTAAT--ATG----------GATTTA
At4g16740c : AGAAGACACTAACGAATGTGCATGTTAAAAATGTGCGAGCACACAAAAACCGGATAGATCGAAACCGATGGGGAGATTTA
At4g16740g : AGAAGACACTAACGAATGTGCATGTTAAAAATGTGCGAGCACACAAAAACCGGATAGATCGAAACCGATGGGGAGATTTA

AF276072.1 : AATCTTAGATCCCTTGGTTTTAGACTCTTGAGACAACATGGATATCATATTCCTCAAG----------------------
At4g16740c : TACGCGACCGCCCTTGAGTTCCGACTCCTAAGGCAACATGGTTTCAGTATCGCACAAG----------------------
At4g16740g : TACGCGACCGCCCTTGAGTTCCGACTCCTAAGGCAACATGGTTTCAGTATCGCACAAGGTTGAAAGCAAAAAAAAAAAAA

AF276072.1 : --------------------------------------------------------------------------------
At4g16740c : --------------------------------------------------------------------------------
At4g16740g : AAAAAAAAAAATCAAAAAGTTATTCACATAAATCCAAATTTTCAGTTTTTAGTTCAAAGTTTCAAAAATGAATTATATAA

AF276072.1 : --------------------------------------------------------------------------------
At4g16740c : --------------------------------------------------------------------------------
At4g16740g : AGTTGGTCTTCAGTTATATATGTCTATATGATGGTTCTGAATTTAACCACATTAATATTGCAATCTCTTGCTGTTGTTAA

AF276072.1 : ---------------------------AGATATTCAAGGACTTTATAGACGTGAATGGAAATTTCAAGGGAGATATCATC
At4g16740c : ------------------------------ATGTTTTTGACGGAAATATTGGAGTTGATTTGGATGATAAAGACATCAAG
At4g16740g : TAACCACATTAATATTGCAATCTCTTGCAGATGTTTTTGACGGAAATATTGGAGTTGATTTGGATGATAAAGACATCAAG

AF276072.1 : AGCATGCTAAATTTGTATGAAGCTTCTTATCATTCAGTAGAGGAGGAAAGTATATTGGATGATGCTAGAGAGTTCACAAC
At4g16740c : GGTATTCTTTCACTATACGAAGCTTCATATCTCTCGACCAGAATCGATACTAAATTGAAAGAGAGCATATACTATACAAC
At4g16740g : GGTATTCTTTCACTATACGAAGCTTCATATCTCTCGACCAGAATCGATACTAAATTGAAAGAGAGCATATACTATACAAC

AF276072.1 : AAAATATTTGAAAGAAACTTTAGAGAATATTGAAGATCAAAATATAGCGTT------------GTTCATAAGTCATGCAT
At4g16740c : AAAACGACTTAGAAAATTTGTGGAGGTAAATAAGAATGAGACCAAATCTTACACTCTTCGAAGGATGGTTATACATGCGT
At4g16740g : AAAACGACTTAGAAAATTTGTGGAGGTAAATAAGAATGAGACCAAATCTTACACTCTTCGAAGGATGGTTATACATGCGT

AF276072.1 : TGGTTTTTCCACTTCATTGGATGGTTCCACGGGTGGAAACAAGTTGGTTTATTGAAGTTTATCCGAAAAAAGTTGGCATG
At4g16740c : TAGAGATGCCGTACCACCGGAGAGTGGGAAGACTAGAAGCAAGATGGTACATAGAAGTGTACGGAGAGAGACACGACATG
At4g16740g : TAGAGATGCCGTACCACCGGAGAGTGGGAAGACTAGAAGCAAGATGGTACATAGAAGTGTACGGAGAGAGACACGACATG

AF276072.1 : AATCCCACGGTGCTTGAGTTTGCGAAACTGGACTTCAACATACTGCAGGCAGTTCACCAAGAAGATATGAAAAAAGCATC
At4g16740c : AACCCTATCTTGCTTGAACTCGCGAAACTTGATTTTAATTTCGTACAAGCTATCCATCAAGACGAGCTCAAATCCCTCTC
At4g16740g : AACCCTATCTTGCTTGAACTCGCGAAACTTGATTTTAATTTCGTACAAGCTATCCATCAAGACGAGCTCAAATCCCTCTC

AF276072.1 : AAG-----------------------------------------------------------------------------
At4g16740c : TAG-----------------------------------------------------------------------------
At4g16740g : TAGGTAAGTTAACTTAAGTAACTGATGATTCGTGGACTTTGAGTAATCATACATGAAATTTGAAAATCAAAAACATATAG

AF276072.1 : --------------------------------------------------------------------------------
At4g16740c : --------------------------------------------------------------------------------
At4g16740g : AGAACATGTTCGTCATATGTTCTTAGAGTACTTTAGTAAACATAAACATGTGTATAACCGATTCAGAAGGTTTGTATTCC

AF276072.1 : --------------------------------------------------------------------------------
At4g16740c : --------------------------------------------------------------------------------
At4g16740g : AACTAAAAAGGATTGAGCAAAAAAAAACCATCATCTCAAGAGACGTGTTAAAAATCTCAAGGTAGAAATGAAAAGAGAAT

AF276072.1 : --------------------------------------------------------------------------------
At4g16740c : --------------------------------------------------------------------------------
At4g16740g : TGCATTACTAATTCATAAACTTATGACTACTCTGTTAAATTGTTTTTAGTTTGGAATTTTCATGATTTAATTTCTCATCA
```

```
AF276072.1 : ------------------------------------------------------------------------------------
At4g16740c : ------------------------------------------------------------------------------------
At4g16740g : TTTATATGTTAAAACTGTGACTTGACCTTCAAGAAAAATCATTATATCTTAAAATTTCTATAGATAATTTCAAAATGTAA

AF276072.1 : ---------------------------------------------ATGGTGGAAAGAAACATGCTGGGAGAAGTTTGGCTTT
At4g16740c : -----------------------------------------TTGGTGGAGCAAGACGGGATTAACAAAACACCTCGATTTC
At4g16740g : TTCTGTCTACATAGTTCATCTTTTTATATGTATGAGACAGTTGGTGGAGCAAGACGGGATTAACAAAACACCTCGATTTC

AF276072.1 : GCTCGTGATCGTTTGGTGGAGAACTTCATGTGGACTGTTGCCGAAAATTACTTGCCTCATTTTCAAACAGGAAGGGGAGT
At4g16740c : GTTAGAGATCGAATAACGGAGGGTTATTTCTCGAGTGTTGGAGTAATGTATGAGCCCGAGTTTGCATATCACCGACAAAT
At4g16740g : GTTAGAGATCGAATAACGGAGGGTTATTTCTCGAGTGTTGGAGTAATGTATGAGCCCGAGTTTGCATATCACCGACAAAT

AF276072.1 : TCTCACAAAGGTTAACGCCATGATAACCACTATCGACGATGTTTATGATGTGTATGGTACTTTGCCTGAACTCGAACTAT
At4g16740c : GCTTACAAAGGTTTTCATGCTCATTACAACTATCGACGATATATACGATATTTATGGCACACTTGAGGAGCTCCAACTAT
At4g16740g : GCTTACAAAGGTTTTCATGCTCATTACAACTATCGACGATATATACGATATTTATGGCACACTTGAGGAGCTCCAACTAT

AF276072.1 : TTACCAACATTGTAAACAG--------------------------------------------------------------
At4g16740c : TCACGACCATAGTTGAAAA--------------------------------------------------------------
At4g16740g : TCACGACCATAGTTGAAAAGTTACTATTAATACCTTGCAAAATAATATCAAACTATTTCACTCTATATCTATTTGCTTAT

AF276072.1 : -----------------------------TTGGGATATCAATGCGATTGATGAACTTCCGGATTATTTGAAAATATGCTTC
At4g16740c : -----------------------------ATGGGATGTGAATCGTCTTGAAGAACTTCCCAACTACATGAAGTTATGTTTT
At4g16740g : TTCTTCAGTGAATCGATATATCATGCAGATGGGATGTGAATCGTCTTGAAGAACTTCCCAACTACATGAAGTTATGTTTT

AF276072.1 : CTTGCGTGCTACAATGCTACCAATGAATTATCATATAACACATTGACAAACAAAGGATTCTTCGTACATCCTTACCTTAA
At4g16740c : CTCTGCCTCGTCAACGAAATCAATCAGATTGGATATTTTGTACTCAGAGATAAAGGGTTTAATGTGATTCCTTACCTCAA
At4g16740g : CTCTGCCTCGTCAACGAAATCAATCAGATTGGATATTTTGTACTCAGAGATAAAGGGTTTAATGTGATTCCTTACCTCAA

AF276072.1 : AAAGGCG--------------------------------------------------------------------------
At4g16740c : AGAATCT--------------------------------------------------------------------------
At4g16740g : AGAATCTGTACGTATTCTCATCATTTTATCTATGTAACAATAGATTTTGAAATATTAATTTGTTGGTTCATTCAAAGTTA

AF276072.1 : ---------------------------------------------------------------------------------
At4g16740c : ---------------------------------------------------------------------------------
At4g16740g : GCTATAAAAATGAATATACACTATAATATACATATATTGTAGTGTGCAGCAATAGATTATCAACCGGAAAGTATATTGTT

AF276072.1 : ---------------------------------------------------------------------------------
At4g16740c : ---------------------------------------------------------------------------------
At4g16740g : TAAGTTTAAGATTTTTTTCGAAGATTTCTTAAAAAACATAAATGAATTTCGTTTTCTTTTCTCATTTTTTTGTCACCAAG

AF276072.1 : ---------------------------------------------------------------------------TGGCA
At4g16740c : ---------------------------------------------------------------------------TGGGC
At4g16740g : ACTTTTTATTAATAAATATGAATATTTCTAAACTTTTTCTCTACATCTCTTGATAACTTAGTTTTTTCTTTTTAGTGGGC

AF276072.1 : GGATTTATGCAACTCTTACATAATTGAAGCTAAATGGTTCAATGATGGATACACACCAACCTTCAACGAGTTCATTGAAA
At4g16740c : AGATATGTGTACAACGTTTTTGAAAGAGGCAAAGTGGTATAAAAGTGGTTACAAACCTAACTTCGAAGAATACATGCAAA
At4g16740g : AGATATGTGTACAACGTTTTTGAAAGAGGCAAAGTGGTATAAAAGTGGTTACAAACCTAACTTCGAAGAATACATGCAAA

AF276072.1 : ATGCATACATGTCAATAGGAATTGCTCCGATCATCAGGCATGCCTATTTGTTAACATTAACTAGTGTTACCGAAGAAGCA
At4g16740c : ATGGTTGGATCTCAAGCTCAGTCCCTACAATACTTCTACACTTGTTCTGTCTCTTATCCGACCAAACCTTAGACATTCTT
At4g16740g : ATGGTTGGATCTCAAGCTCAGTCCCTACAATACTTCTACACTTGTTCTGTCTCTTATCCGACCAAACCTTAGACATTCTT

AF276072.1 : TTGCAACACATAGAAAGAGCTGAAAGTATGATTCGCAATGCATGCCTAATTGTGCGACTCACTAATGATATGGGCACATC
At4g16740c : GGCTCCTACAA---------TCACTCTGTAGTTCGAAGCTCCGCCACCATCCTCCGTCTCGCTAACGATCTCGCCACTTC
At4g16740g : GGCTCCTACAA-------TCACTCTGTAGTTCGAAGCTCCGCCACCATCCTCCGTCTCGCTAACGATCTCGCCACTTCTT

AF276072.1 : ATCTG----------------------------------------------------------------------------
At4g16740c : TTCGG----------------------------------------------------------------------------
At4g16740g : CGGTCAGTCATGGTTTCACAACGTACAATACTGTAAATAATTTATTGACAGATCAAAGGCGATTATATCTAAGCAAACTT

AF276072.1 : ---------------------------------------------------------------------------------
At4g16740c : ---------------------------------------------------------------------------------
At4g16740g : TTTCAGACTAAATAAACAAAGTCAACCAACATATGGGGACTCACACATGTGTTTCAAAGTTATGCTCAAAGCGGTCCTAT

AF276072.1 : ------------------------ATGAGCTTGAAAGAGGTGATATTCCAAAATCAATCCAGTGCTATATGCACGAAAGTG
At4g16740c : ------------------------AGGAATTAGCGAGAGGCGACACTATGAAATCCGTACAATGTCACATGCATGAAACTG
At4g16740g : AAATATAGTTATTTTAACAGGAGGAATTAGCGAGAGGCGACACTATGAAATCCGTACAATGTCACATGCATGAAACTGGA

AF276072.1 : GTGCTACTGAAATGGAAGCACGAGCGTATATAAAACAGTTCATCGTCGAGACATGGAAGAAACTGAACAAAGAACGGCAA
At4g16740c : GAGCTTCGGAGGCAGAGTCACGCGCGTACATTCAAGGAATTATCGGTGTGGCTTGGGATGACTTAAACATGGAGA-----
At4g16740g : GCTTCGGAGGCAGAGTCACGCGCGTACATTCAAGGAATTATCGGTGTGGCTTGGGATGACTTAAACATGGAGA------A

AF276072.1 : GAAATTGGTTCTGAATTTCCGCAAGAGTTCGTTGATTGTGTTATAAACCTTCCTAGAATGGGTCATTTCATGTATACCGA
At4g16740c : -AAAAGAGTTGTAGGCTACATCAAGGTTTCCTAGAAGCTGCGGCTAATCTTGGACGTGTGGCTCAGTGCGTTTATCAGTA
At4g16740g : AAAGAGTTGTAGGCTACATCAAGGTTTCCTAGAAGCTGCGGCTAATCTTGGACGTGTGGCTCAGTGCGTTTATCAGTACG

AF276072.1 : TGGAGACAAACATGGTAAACCCGACATGTTCAAGCCGTATGTATTTTCATTGTTTGTTAATCCAATCTAG
At4g16740c : CGGTGATGGCCATGGCTGTCCTGACAAGGCTAAGACC-------------------GTCAATCATGTCCGG
At4g16740g : GTGATGGCCATGGCTGTCCTGACAAGGCTAAGACC------------------GTCAATCATGTCCGGTC
```
